# Frequent *PTEN *genomic alterations and activated phosphatidylinositol 3-kinase pathway in basal-like breast cancer cells

**DOI:** 10.1186/bcr2204

**Published:** 2008-12-03

**Authors:** Bérengère Marty, Virginie Maire, Eléonore Gravier, Guillem Rigaill, Anne Vincent-Salomon, Marion Kappler, Ingrid Lebigot, Fathia Djelti, Audrey Tourdès, Pierre Gestraud, Philippe Hupé, Emmanuel Barillot, Francisco Cruzalegui, Gordon C Tucker, Marc-Henri Stern, Jean-Paul Thiery, John A Hickman, Thierry Dubois

**Affiliations:** 1 Département de Transfert, Institut Curie, 26 rue d'Ulm, 75005 Paris, France; 2 Département de Biostatistiques, Institut Curie, 26 rue d'Ulm, 75005 Paris, France; 3 INSERM U900, Institut Curie, 26 rue d'Ulm, 75005 Paris, France; 4 Service de Pathologie, Institut Curie, 26 rue d'Ulm, 75005 Paris, France; 5 CNRS UMR144, Institut Curie, 26 rue d'Ulm, 75005 Paris, France; 6 Ecole des Mines de Paris, 77300 Fontainebleau, France; 7 Unité de Mathématiques et Informatique Appliquées, UMR518, AgroParisTech/INRA, 75005 Paris, France; 8 Institut de Recherches Servier, 125 Chemin de Ronde, 78290 Croissy sur Seine, France; 9 INSERM U830, Institut Curie, 26 rue d'Ulm, 75005 Paris, France; 10 Current address: Institute of Molecular and Cell Biology, 61 Biopolis Drive (Proteos), 138673 Singapore

## Abstract

**Introduction:**

Basal-like carcinomas (BLCs) and human epidermal growth factor receptor 2 overexpressing (HER2+) carcinomas are the subgroups of breast cancers that have the most aggressive clinical behaviour. In contrast to HER2+ carcinomas, no targeted therapy is currently available for the treatment of patients with BLCs. In order to discover potential therapeutic targets, we aimed to discover deregulated signalling pathways in human BLCs.

**Methods:**

In this study, we focused on the oncogenic phosphatidylinositol 3-kinase (PI3K) pathway in 13 BLCs, and compared it with a control series of 11 hormonal receptor negative- and grade III-matched HER2+ carcinomas. The two tumour populations were first characterised by immunohistochemistry and gene expression. The PI3K pathway was then investigated by gene copy-number analysis, gene expression profiling and at a proteomic level using reverse-phase protein array technology and tissue microarray. The effects of the PI3K inhibition pathway on proliferation and apoptosis was further analysed in three human basal-like cell lines.

**Results:**

The PI3K pathway was found to be activated in BLCs and up-regulated compared with HER2+ tumours as shown by a significantly increased activation of the downstream targets Akt and mTOR (mammalian target of rapamycin). BLCs expressed significantly lower levels of the tumour suppressor PTEN and PTEN levels were significantly negatively correlated with Akt activity within that population. PTEN protein expression correlated significantly with *PTEN *DNA copy number and more importantly, reduced *PTEN *DNA copy numbers were observed specifically in BLCs. Similar to human samples, basal-like cell lines exhibited an activation of PI3K/Akt pathway and low/lack PTEN expression. Both PI3K and mTOR inhibitors led to basal-like cell growth arrest. However, apoptosis was specifically observed after PI3K inhibition.

**Conclusions:**

These data provide insight into the molecular pathogenesis of BLCs and implicate the PTEN-dependent activated Akt signalling pathway as a potential therapeutic target for the management of patients with poor prognosis BLCs.

## Introduction

Gene expression profiling has enabled the identification of five subgroups of breast cancer characterised by different clinical outcomes and responses to therapy [1-10]. Among them, basal-like carcinomas (BLC) and human epidermal growth factor receptor 2 overexpressing (HER2+) carcinomas are associated with the worst prognosis [6,10,11]. BLCs are highly proliferative, genetically unstable, poorly differentiated, often grade III carcinomas [12,13] and preferentially metastase in the brain and lungs [14]. They are identified by immunohistochemistry as triple negative (lack of HER2 and oestrogen/progesterone receptor (ER/PR) expression) and positive for basal cytokeratins (CK5/6 and/or CK14) and/or epidermal growth factor receptor (EGFR) expression [8,15]. BLCs represent about 15% of cases of breast cancer and appear to be prevalent in pre-menopausal African American woman (39%) [16].

Patients with BLCs are treated exclusively with conventional therapy. Although they show high rates of objective initial response, the majority of patients do not have a complete, prolonged response, and they have a poorer prognosis than those within other breast tumour subgroups [12,13]. In contrast to HER2+ carcinomas treated with targeted therapy such as anti-HER2 [17], there is no available targeted therapy for BLCs. However, in patients with triple-negative breast cancer, some treatments are in preclinical trials, such as Dasatinib, a Src tyrosine kinase inhibitor, Cetuximab or Bevacizumab, which target EGFR and vascular endothelial growth factor, respectively [18]. Little is known about the pathogenesis of BLCs in spite of the recent genome and transcriptome microarray profiling [14,15,19,20]. Proteomics in tandem with genomic/transcriptomic analysis is essential to clarify the molecular pathology of BLCs and to discover druggable targets [21,22].

In order to identify such targets, we are exploring the phosphoproteome of BLCs to highlight deregulated signalling pathways. In this report, we have investigated the oncogenic phosphatidylinositol 3-kinase (PI3K) pathway in BLCs and compared it with that of HER2+ carcinomas in which it is known to be up-regulated [23-25]. Phosphatidylinositol-3,4,5-trisphosphate (PIP3) is an important lipid second messenger in tumourigenesis, in particular by activating Akt, which binds to membrane-associated PIP3 through its plekstrin homology domain, and other signalling molecules involved in a variety of cellular events, such as survival, proliferation, cell motility and invasion [26]. PI3K is activated downstream of extracellular signals and phosphorylates phosphatidylinositol-4,5-bisphosphate to generate PIP3. The tumour suppressor PTEN (phosphatase and tensin homologue deleted on chromosome 10) catalyses the opposite reaction, thereby reducing the pool of PIP3, inhibiting growth and survival signals, and suppressing tumour formation [27,28]. The PI3K signalling pathway is frequently deregulated in human solid tumours including breast cancers through Akt1 or PIK3CA (catalytic subunit of PI3K) mutations, HER2 overexpression and PTEN loss or mutation [24,25,29-34].

In this report, we demonstrate that the PI3K pathway is activated in BLCs. The PI3K pathway was up-regulated in BLCs compared with HER2+ carcinomas as shown by a significant increased activation of downstream targets such as Akt and mTOR (mammalian target of rapamycin). We also describe the molecular mechanism leading to this PI3K pathway activation, which occurs through a low PTEN protein expression that was found to be associated with genomic alterations at the *PTEN *locus, specifically in BLCs. In addition, we observed that basal-like cell lines exhibited an activation of Akt and a low/lack of PTEN expression. The exposure of basal-like cell lines to PI3K or mTOR inhibitors led to cell growth arrest. However, apoptosis was detected when PI3K, but not mTOR, was inhibited. Altogether, our data demonstrate a PTEN-dependent up-regulated PI3K pathway in BLCs and suggest this pathway as a therapeutic target for patients with poor prognosis BLCs.

## Materials and methods

### Immunohistochemistry

Twenty-four tumours were obtained from patients treated at the Curie Institute (Biological Resource Centre, Paris, France). Immunohistochemistry was performed as previously described [35]. Tumours contained between 50% and 90% tumour cells revealed by haematoxylin-eosin-safran (HES) staining.

For phospho-Akt (S473) staining, tissue microarrays (TMA) containing alcohol, formalin and acetic acid (AFA)-fixed paraffin-embedded tissue were made. For each biopsy, three representative tumour areas and one peritumoural tissue were carefully selected from a HES-stained section of a donor block. Using a specific arraying device (Manual Tissue Arrayer; Beecher Instruments, Sun Prairie, WI, USA) core cylinders of 1 mm in diameter were punched from each of those four areas and placed into recipient paraffin blocks. Sections of 3 μm were cut, placed onto positively charged slides (capillary gap microscope slides, Dako REAL, Dako, Trappes, France) and dried at 58°C for one hour. Sections were deparaffinised in toluene and hydrated in graded alcohol. Antigen retrieval was performed in 10 mM sodium citrate (pH 6.10) for 20 minutes at 95°C. Sections were then cooled for 20 minutes at room temperature. Endogenous biotins were blocked by Biotin blocking system (Dako, Trappes, France).

After washes in PBS-Tween buffer, endogenous peroxidase activity was quenched with 3% hydrogen peroxide for 5 minutes then rinsed in distilled water. Each tissue section was blocked with a solution of PBS (pH 7.4) containing 1% of BSA and 1.4% of normal horse serum for 5 minutes, followed by an overnight incubation at 4°C with primary antibody against phospho-Akt (S473). After washes, slides were incubated with rabbit biotinylated antibody (Jackson Immunoresearch, Interchim, Clichy, France) for 30 minutes. Immunostaining was revealed using the Vectastain ABC peroxidase system (Vector Laboratories, Abcys, Paris, France) using diaminobenzidine as a chromogen. Slides were counter-stained with haematoxylin before mounting. The reactions were carried out using an automated stainer (LabVision, Thermo Scientific, Microm France, Francheville, France) except for the primary antibody. Omission of the primary antibody was used as a negative control. Immunohistochemistry conditions were first optimised using cell pellets from cell lines known to be positive or negative for phospho-Akt staining.

**Figure 1:**
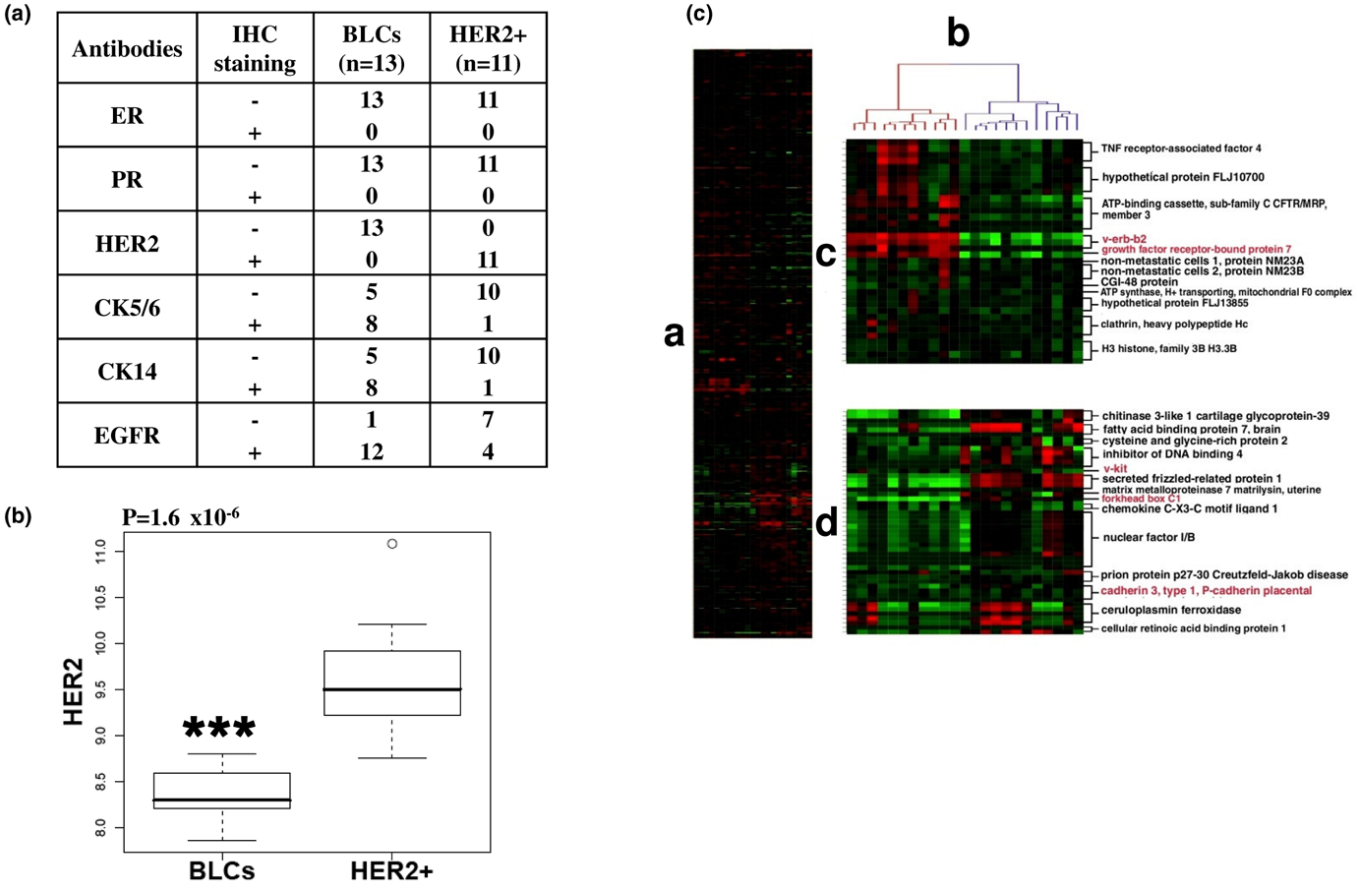
**Characterisation of basal-like carcinomas (BLCs) and human epidermal growth factor receptor overexpressing (HER2+) carcinomas from human biopsies**. (a) Selection and characterisation of human samples by immunohistochemistry (IHC). Thirteen BLCs were first selected as grade III triple-negative ductal carcinomas (negative for oestrogen receptor (ER), progesterone receptor (PR) and HER2 expression) and then characterised for cytokeratin 5/6 (CK5/6), CK14 and epidermal growth factor receptor (EGFR) staining. The control series was composed of 11 hormone receptor negative- and grade III-matched HER2+ carcinomas. Tumours contained between 50% and 90% tumour cells revealed by haematoxylin-eosin-safran staining. (b) Higher HER2 protein expression in HER2+ carcinomas compared with BLCs. The box plot illustrates the expression of total HER2 protein expression measured by reverse phase protein array (RPPA) in human BLCs and HER2+ carcinomas. An outlier is present within the HER2+ population (open circle). The y axis represents logarithmic transformed HER2 relative quantification. The p value (*** p < 0.001) is represented (Mann-Whitney test). Data are representative of three separate RPPA experiments. (c) Tumours selected by immunohistochemistry clustered according to their gene expression signature. A hierarchical clustering was performed on the intrinsic/UNC genes as described [46]. (i) Overview of complete cluster diagram. Each row represents a gene and each column a human tumour. Black is for no change, red for up-regulation and green for down-regulation of gene expression. (ii) Experimental sample-associated dendrogram. Red dendrogram branch represents HER2+ carcinomas and blue designs basal-like carcinomas. (iii) HER2+ signature. A HER2+ expression cluster was observed and contained multiples genes from the 17q11 amplicon including HER2 and growth factor receptor-bound protein 7 (GRB7) (typed in red) as previously described [46]. (iv) Basal-like signature. A basal-like expression cluster was found and contained genes previously identified to be characteristic of basal epithelial cells such as v-kit, FOXC1 and P-cadherin (written in red) [46].

Positive nuclear staining for ER and PR were recorded in accordance with standardised guidelines, using 10% as the cut-off for ER- and PR-positive cells. For HER2, only staining of membranes was considered with a 30% cut-off as recommended [36]. The cut-off for CK5/6, CK14 and EGFR positivity was 10% of stained cells (weak or strong) for the results shown in Figure 1a.

EGFR (clone 31G7, 1:40 dilution, Zymed, Invitrogen, Cergy-Pontoise, France), CK5/6 (clone D5/16B4, 1:50 dilution, Dako, Trappes, France), CK14 (clone LL002, prediluted, Biogenex, San Ramon, CA, USA) and phospho-Akt (S473) (clone 736E11, 1:50 dilution, Cell Signaling Technology, Ozyme, Saint Quentin en Yveline, France) antibodies were used.

### Tumour lysis

Frozen tumours were incubated with a lysis buffer containing 50 mM Tris (pH 6.8), 2% sodium dodecyl sulfate (SDS), 5% glycerol, 2 mM 1,4-dithio-DL-threitol (DTT), 2.5 mM ethylenediaminetetraacetic acid, 2.5 mM ethylene glycol tetraacetic acid, 2 mM sodium orthovanadate, 10 mM sodium fluoride and a cocktail of protease (Roche, Meylan, France) and phosphatase (Pierce, Perbio, Brebières, France) inhibitors. Homogenisation was obtained using a TissueLyser (Qiagen, Courtaboeuf, France) with stainless steel beads 5 mm in diameter (Qiagen, Courtaboeuf, France) for two to three minutes at 30 Hz. Lysates were boiled at 100°C for 10 minutes to inactivate proteases and phosphatases. Protein concentration was determined using the BCA Protein Assay Kit-Reducing Agent Compatible (Pierce, Perbio, Brebières, France). Lysates were then stored at -80°C.

### Reverse phase protein array

We developed a robust reverse phase protein array (RPPA) technology allowing the printing of very small quantities of protein (about 1 ng per spot) convenient for the analysis of minimal quantities of biopsy material. This miniaturised dot-blot technology is based on robotic printing of a large number of different cell/tissue lysates onto nitrocellulose bound to histology slides and the analysis of proteins of interest with highly specific antibodies [37,38]. Five two-fold serial dilutions were made from each lysate in 96-well plates (conical bottom, 50 μl/well) and spotted in triplicates onto nitrocellulose-coated glass slides (FAST slides, Whatman, Schleicher & Schuell, Maidstone, Kent, UK) by using a MicroGrid Compact arrayer (BioRobotics, Dutscher Scientific Instrumentation, Brumath, France) with SMP3XB pins (Tip diameter = 75 μm, volume-spot = 1.2 nl; Telechem, Proteigene, Saint Marcel, France).

To avoid evaporation during spotting, the humidity was kept at about 50% to 60% in the array chamber with a humidification control unit. After printing, slides coated with two nitrocellulose pads were incubated with avidin, biotin and peroxydase blocking reagents (Dako, Trappes, France) before saturation with TBS containing 0.1% Tween-20 and 5% BSA (TBST-BSA). Each pad was then probed overnight at 4°C with primary antibodies (or without primary antibodies, for negative controls) at the appropriate dilution in TBST-BSA. After washes with TBST, arrays were probed with horseradish peroxidase secondary antibodies (Jackson ImmunoResearch Laboratories, Interchim, Clichy, France) diluted in TBST-BSA for one hour at room temperature. To amplify the signal, slides were incubated with Bio-Rad Amplification Reagent (BAR solution) supplied in the Western blot amplification module (Bio-Rad, Marnes la Coquette, France) for 10 minutes at room temperature. The arrays were washed with TBST containing 10% dimethyl sulfoxide (DMSO) for two minutes, then with TBST. To detect the bound biotin, slides were probed with Cy5-Streptavidin (Jackson ImmunoResearch Laboratories, Interchim, Clichy, France) diluted in TBST-BSA for one hour at room temperature. The processed slides were scanned using a GenePix 4000B microarray scanner (Molecular Devices, Saint Grégoire, France). Double staining was performed to quantify actin expression for the normalisation between samples using anti-beta-actin primary antibodies (Sigma-Aldrich, Saint Quentin Fallavier, France) and Cy3 secondary antibodies (Jackson ImmunoResearch Laboratories, Interchim, Clichy, France).

Specificity of each primary antibody used in this study was first validated by Western blotting on several cell and tumour lysates (not shown). Optimal dilution was determined for each antibody with different cell lysates using specific software developed at the Curie Institute with the following criteria: signal away from the negative control without saturation and correlation with Western blotting. Spot detection and quantification were determined with MicroVigene software (VigeneTech Inc, Carlisle, MA). Akt phospho-Akt (S473), PTEN and stathmin antibodies (Cell Signaling Technology, Ozyme, Saint Quentin en Yveline, France) were utilised at a dilution of 1:1000, 1:250, 1:200 and 1:100, respectively. HER2 antibodies (clone MS-432-P1, Ab11) used at 1:500 dilution were from Lab Vision (Interchim, Clichy, France). mTOR expression and phosphorylation was not examined by RPPA due to the poor specificity of mTOR antibodies.

### Western-blotting

Tissue lysates (10 μg/lane) were loaded onto 10% or four 12% Bis-Tris Criterion XT gels (Bio-Rad, Marnes la Coquette, France) and migration was performed using MOPS buffer (Bio-Rad, Marnes la Coquette, France). Proteins were then transferred to nitrocellulose (Bio-Rad, Marnes la Coquette, France). Membranes were saturated with TBST-BSA and then incubated overnight at 4°C with primary antibodies at the appropriate dilution in TBST-BSA. After washes, membranes were incubated with horseradish peroxidase secondary antibodies (Jackson ImmunoResearch Laboratories, Interchim, Clichy, France) for one hour at room temperature. Bound antibodies on immunoblots were visualised on membranes with a chemoluminescent detection system (ECL; Amersham Pharmacia Biotech, Orsay, France). Quantification was performed using a LAS-3000 Luminescent Image analyser and Image Gauge software (Fuji, FSVT, Courbevoie, France). Actin was detected for normalisation between samples using anti-beta-actin primary antibodies at the dilution of 1:5000 (Sigma-Aldrich, Saint Quentin Fallavier, France). Akt, phospho-Akt (S473), mTOR, phospho-mTOR (S2448), PTEN and cleaved-PARP antibodies (Cell Signaling Technology, Ozyme, Saint Quentin en Yveline, France) were used at 1:1000 dilution. HER2 antibodies (clone MS-432-P1, Ab11) were used at a 1:500 dilution (Lab Vision, Interchim, Clichy, France).

### DNA and RNA microarray analysis

DNA and RNA were purified as described [20]. For genomic arrays, Affymetrix GeneChip Human Mapping 100 K was normalised and analysed using ITALICS (ITerative and Alternative normaLIzation and Copy number calling for affymetrix Snp arrays) algorithm [39]. The segmentation of the genomic profile was performed using GLAD (Gain and Loss Analysis of DNA) software [40]. The forceGL parameter was set to 0.28. Single nucleotide polymorphisms with smoothing value lower and greater than 2 ± 0.28 were considered as loss and gain, respectively. After RNA quality control, 12 of the 13 BLCs and the 11 HER2+ carcinomas were hybridised onto U133 plus 2.0 Affymetrix chips. Transcriptomic data were normalised using GC-RMA [41]. Raw and normalised transcriptomic data are publically available at Gene Expression Omnibus (Accession number: [GSE13787]) and at the Curie Institute microarray dataset repositories [42].

### Cell culture

The cell lines were obtained from the American Type Culture Collection (LGC Promochem, Molsheim, France) and from the European Collection of Animal Cell Cultures (Sigma-Aldrich, Saint Quentin Fallavier, France). HCC38 and HCC1937 were maintained in RPMI-1640 with 10% FBS, 1.5 g/L sodium bicarbonate, 10 mM Hepes and 1 mM sodium pyruvate. BT20 were cultured in Eagle's minimal essential medium containing 10% FBS, 1.5 g/L sodium bicarbonate, 0.1 mM non-essential amino acids and 1 mM sodium pyruvate. MDA-MB-468 were grown with RPMI with 10% FBS. MDA-MB-453 were cultured without carbon dioxide in Leibovitz's L-15 medium containing 10% FBS and 10 mM HEPES. SKBr3 were grown with McCoy5a containing 10% FBS and A431 with Eagle's minimal essential medium containing 10% FBS and 0.1 mM non-essential amino acids. A431 cells were either or not stimulated with 50 ng/ml EGF for five minutes after overnight serum starvation. Lysates were prepared at 60% to 90% cell confluency and analysed by Western blotting.

### Cell proliferation assay

To test the effect of LY294002 and rapamycin on cell proliferation, cells were seeded into 96-well plates at a density determined on the basis of the growth characteristics of each cell line (750 cells/well for MDA-MB-468 and HCC1937; 1500 cells/well for BT20). Forty-eight hours later, cells (triplicate wells) were treated for seven days with varying concentration of LY294002 (Sigma-Aldrich, Saint Quentin Fallavier, France), rapamycin (Cell Signaling Technology, Ozyme, Saint Quentin en Yveline, France) or DMSO (Sigma-Aldrich, Saint Quentin Fallavier, France) as a control. LY094002 concentrations tested were 0.39, 0.78, 1.56, 3.12, 6.25, 12.5, 25 and 50 μM. Rapamycin concentrations analysed were 0.49, 0.98, 1.95, 3.91, 7.81, 15.62, 31.25, 62.5, 125 and 250 nM.

The relative percentages of metabolically active cells compared with untreated controls were determined on the basis of mitochondrial conversion of 3-(4,5-dimethylthiazol-2-yl)-2,5 diphenyltetrazolium bromide (MTT) to formazine using a MTT assay. To each well, 15 μL of MTT (5 mg/mL in PBS) was added. After four hours incubation at 37°C, floating plus adherent cells were lysed by the addition of 10% SDS in 10 mM hydrochloric acid. The absorbance was measured at the wavelength of 540 nm (Infinite 200, Tecan, Lyon, France) and results are presented as the percentage of control cell growth inhibition obtained from no treated cells grown in the same culture plate. The IC_50_s were determined on the basis of the dose-response curves.

### Apoptosis assays

Cells were harvested and seeded in 96-well plates (10 000 cells/well). After overnight growth, cells were treated in triplicate with various concentrations of LY294002, rapamycin or DMSO as a control. Twenty-four hours later, apoptosis was determined by caspase 3/7 activation and by the detection of PARP cleavage that serves as a marker of cells undergoing apoptosis. Caspase activity was determined using Caspase-Glo 3/7 luminescent assay (Promega, Charbonnières-les-bains, France) according to the manufacturer's instructions. Results are presented as caspase 3/7 activity normalised by caspase 3/7 activity from vehicle-treated cells. For PARP cleavage, Western blot was performed using whole protein lysates of floating plus adherent cells. Blots were incubated with a specific cleaved-PARP antibody (Cell Signaling Technology, Saint Quentin en Yveline, France).

### Statistical analysis

As data did not display a normal distribution, a non-parametric test was performed. Mann-Whitney test was used to assess differential expression of a protein between the two groups (BLCs and HER2+). The R software v2.4.0 was used for statistical analyses [43]. A Spearman correlation test was performed to estimate a rank-based measure of association between two parameters. Values were log transformed. p values under 5% were considered significant. For the apoptosis assays, p values were calculated using Student's *t *test.

## Results and discussion

### Tumour selection and characterisation

The PI3K pathway was examined in two populations of highly proliferative, grade III, hormone receptor-negative invasive breast carcinomas. We chose this comparison, rather than that of BLCs with normal tissue, to compare two types of proliferating cells, avoiding a comparison with a largely differentiated, quiescent population. Thirteen BLCs were selected by immunohistochemistry as triple-negative ductal carcinomas (lack of ER, PR and HER2 staining) that expressed CK5/6 and/or CK14 and/or EGFR (Figure 1a). The comparison series was composed of 11 patients with ER-negative/PR-negative and HER2+ tumours (Figure 1a). CK5/6 was expressed in 61.5% BLCs (8 of 13) and 9.1% HER2+ (1 of 11) (Figure 1a). Similarly, CK14 was expressed more in the same BLCs (61.5%) than in HER2+ (9.1%) (Figure 1a). EGFR was detected in 92.3% BLCs (12 of 13) and 36.4% HER2+ (4 of 11) (Figure 1a), in agreement with previous studies showing EGFR expression in most BLCs and in HER2+ carcinomas [8,44,45]. Expectedly, RPPA analysis confirmed a significantly higher HER2 protein expression in HER2+ carcinomas compared with BLCs (p = 1.6 × 10^-6^) (Figure 1b). Similar results were observed by Western blotting and significantly correlated with those obtained by RPPA [see Additional data file 1]. Of note, some BLCs carcinomas expressed HER2 protein but at lower levels than those observed in HER2+ carcinomas. In addition, these data indicated that RPPA technology could be useful to measure in a quantitative manner the expression of HER2 protein in human samples. Gene expression microarray analysis confirmed that the tumours clustered according to basal-like and HER2+ signatures [46] (Figure 1c). Therefore, the two breast cancer populations were accurately characterised and the subtypes identified by immunohistochemistry corresponded to the gene expression classification.

### Activated PI3K pathway in basal-like breast cancer

**Figure 2:**
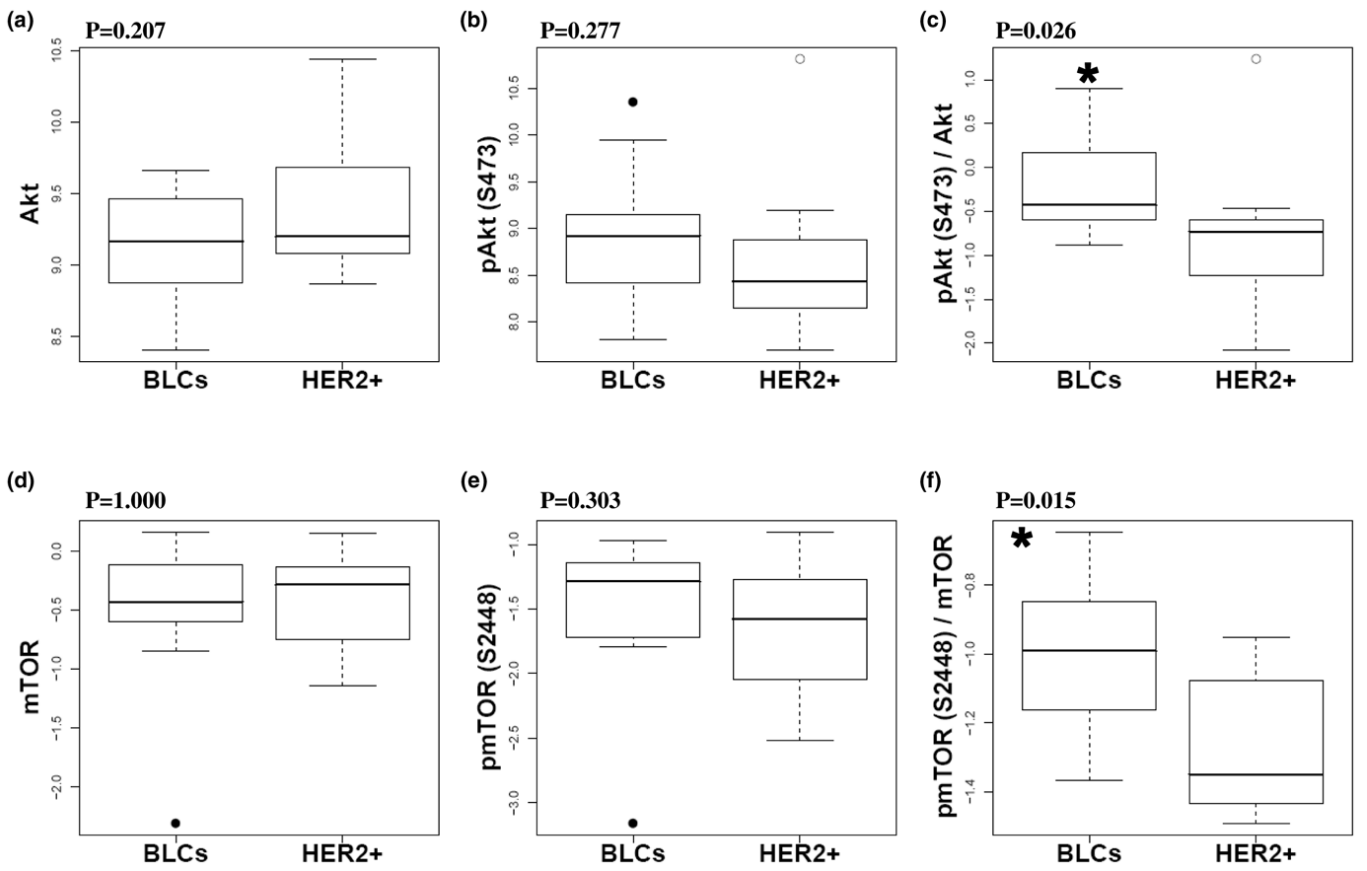
**Up-regulated phosphatidylinositol 3-kinase (PI3K) signalling pathway in human basal-like breast cancers**. Akt is activated in basal-like carcinomas (BLCs). The expression of (a) total Akt and (b) phosphorylated/active form of Akt (phospho-Akt (S473)) were measured by reverse phase protein array (RPPA) as well as Akt activity determined as the (c) ratio 'phospho/total' in human BLCs and human epidermal growth factor receptor overexpressing (HER2+) carcinomas. mTOR is activated in BLCs. Box plots show the (d) expression of mTOR and (e) its form phosphorylated via the PI3 kinase/Akt signalling pathway (phospho-mTOR (S2448)) determined by Western blotting as well as (f) mTOR activity (phospho/total ratio) in human BLCs and HER2+ carcinomas. Outliers are shown for BLCs (solid circles) and HER2+ carcinomas (open circles). The y axes represent logarithmic transformed relative quantifications. p values (* p < 0.05) are represented (Mann-Whitney test). Data are representative of four and two separate experiments for RPPA and Western-blot, respectively.

Proteomic analysis was then continued by RPPA allowing analysis of a very limited amount of sample from biopsies [33,37,38]. Akt was expressed at similar levels in BLCs and HER2 carcinomas (Figure 2a) whereas the phosphorylated and active form of Akt (S473) tended to be expressed more in BLCs although not in a significant manner (Figure 2b). Akt activity, defined as the phospho/total ratio, was significantly increased in BLCs compared with HER2+ population (p = 0.026) (Figure 2c). Similar data, significantly correlated with RPPA data, were obtained by Western blotting [see Additional data file 2] and were in agreement with those showing an activation of Akt within a population of eight triple-negative carcinomas [47].

Our data further revealed that Akt was more active in BLCs compared with HER2+ carcinomas where Akt is known to be activated through HER2 overexpression [23-25]. We verified by immunohistochemistry of both BLCs and HER2+ carcinomas that the active form of Akt was expressed in tumour cells, with a plasma membrane localisation observed in tumours showing strong phospho-Akt immunoreactivity [see Additional data file 3]. We also examined the phosphorylation status of the target of rapamycin, mTOR, particularly at the S2448 residue known to be phosphorylated through PI3K/Akt signalling pathway activation. mTOR was expressed at similar levels in the two breast populations but was significantly more active (phospho/total ratio) in BLCs than in HER2+ carcinomas (p = 0.015) (Figure 2d,e,f), where mTOR has been shown to be activated [48]. The PI3K pathway was up-regulated in BLCs compared with HER2+ as shown by the significant activation of downstream targets such as Akt and mTOR.

### Lower PTEN expression in basal-like breast cancer compared to HER2+ carcinomas

**Figure 3:**
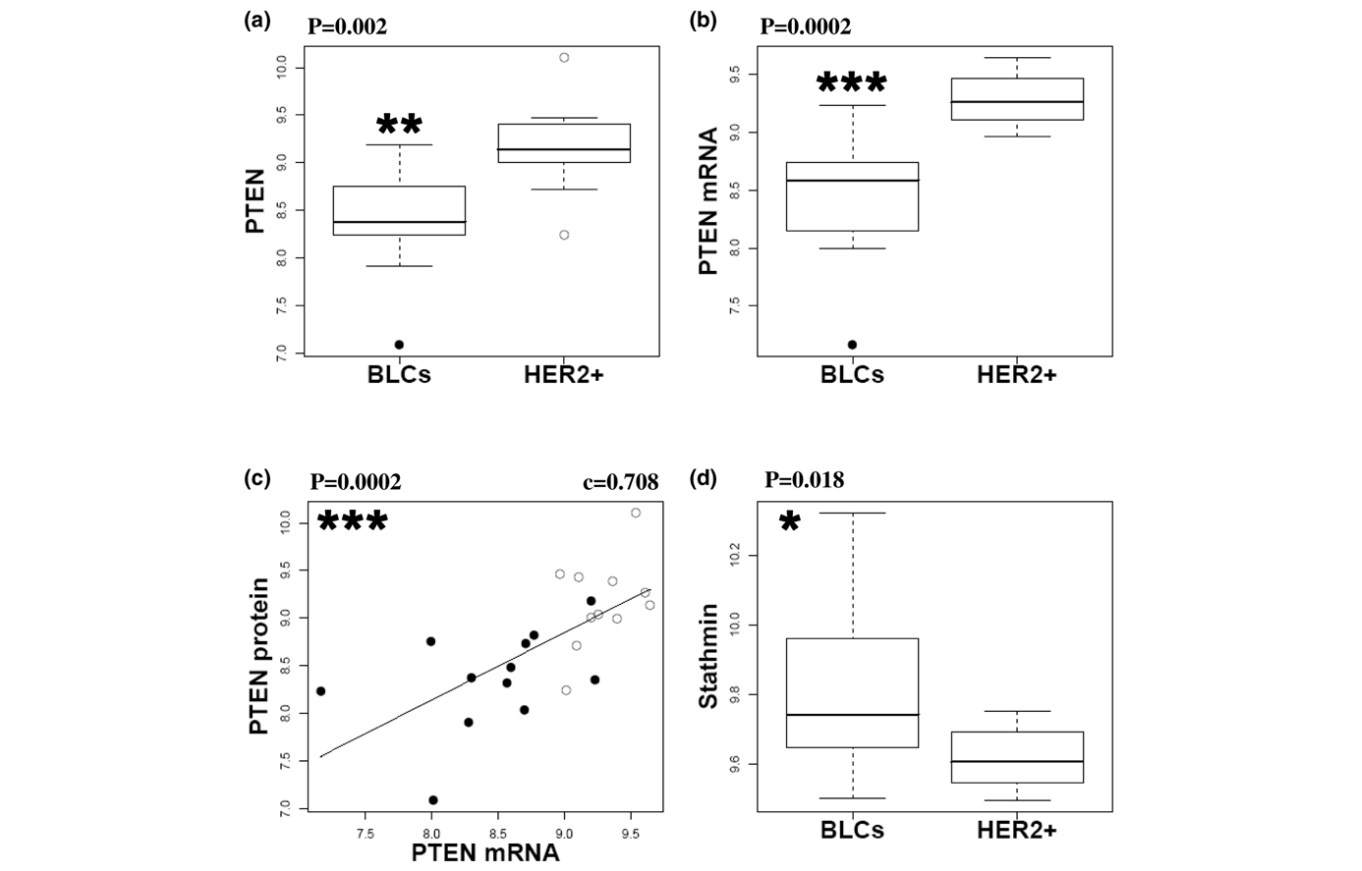
**Lower phosphatase and tensin homolog deleted on chromosome 10 (PTEN) expression in human basal-like cancers (BLCs) compared with human epidermal growth factor receptor overexpressing (HER2+) carcinomas**. (a) Lower PTEN protein levels in BLCs compared with HER2+ carcinomas. PTEN protein level was quantified by reverse phase protein array (RPPA). Outliers are shown for BLCs (solid circles) and HER2+ carcinomas (open circles). Data are representative of four separate experiments. (b) Lower mRNA PTEN level (probeset 225363_at) in BLCs compared with HER2+ carcinomas. An outlier is present in BLC population (solid circles). (c) Correlation between PTEN protein measured by RPPA and PTEN messenger in the entire tumour population. Linear regression, Spearman correlation c and p value are presented. BLCs (solid circles) and HER2+ carcinomas (open circles) are shown. (d) Stathmin is overexpressed in BLCs compared with HER2+ carcinomas. Box plots indicate the levels of stathmin protein measured by RPPA within the two populations. Data are representative of four separate experiments. P values are shown (a,b,d: Mann-Whitney test). * p < 0.05, ** p < 0.01, *** p < 0.001. Protein and mRNA relative quantifications were logarithmic transformed.

We then attempted to characterise the molecular mechanism(s) leading to Akt activation in BLCs. We evaluated PTEN expression because its loss has been associated with ER negative [24,49,50] and CK5/14-positive breast cancer [34]. RPPA analysis highlighted a lower expression of PTEN protein in BLCs compared with HER2+ carcinomas in a significant manner (p = 0.002) (Figure 3a). Similar data were obtained when PTEN was detected by Western blotting and significantly correlated with RPPA data [see Figures a and b in Additional data file 4]. So far, we failed to estimate PTEN level by immunohistochemistry, possibly because of the PTEN antibodies we tested and/or the AFA fixation of tissues. Lower PTEN expression in BLCs was also detected at the mRNA level (p = 0.0002) (Figure 3b).

In agreement with a previous report with PTEN protein levels measured by immunohistochemistry [49], PTEN mRNA and protein levels were well correlated (p = 0.0002) (Figure 3c) [see Figure c in Additional data file 4], indicating that we could estimate PTEN protein levels from transcriptomic analysis. Our analysis of published data [51] showed that lower PTEN mRNA levels in BLCs compared with normal samples (p = 0.003, Mann-Whitney test, data not shown), suggesting lower PTEN protein levels in BLCs compared with normal tissues. We examined the expression of stathmin, which has recently been shown to be overexpressed in low PTEN expressing breast cancers [49]. In accordance with these published observations, stathmin protein was overexpressed in BLCs compared with HER2+ carcinomas (p = 0.018) (Figure 3d). Stathmin therefore represents a potential marker for PTEN-dependent PI3K pathway activation [49]. Altogether, transcriptomic and proteomic analyses highlighted low expression of PTEN in BLCs.

### Genomic alterations at the *PTEN *tumour suppressor gene in basal-like breast cancer

**Figure 4:**
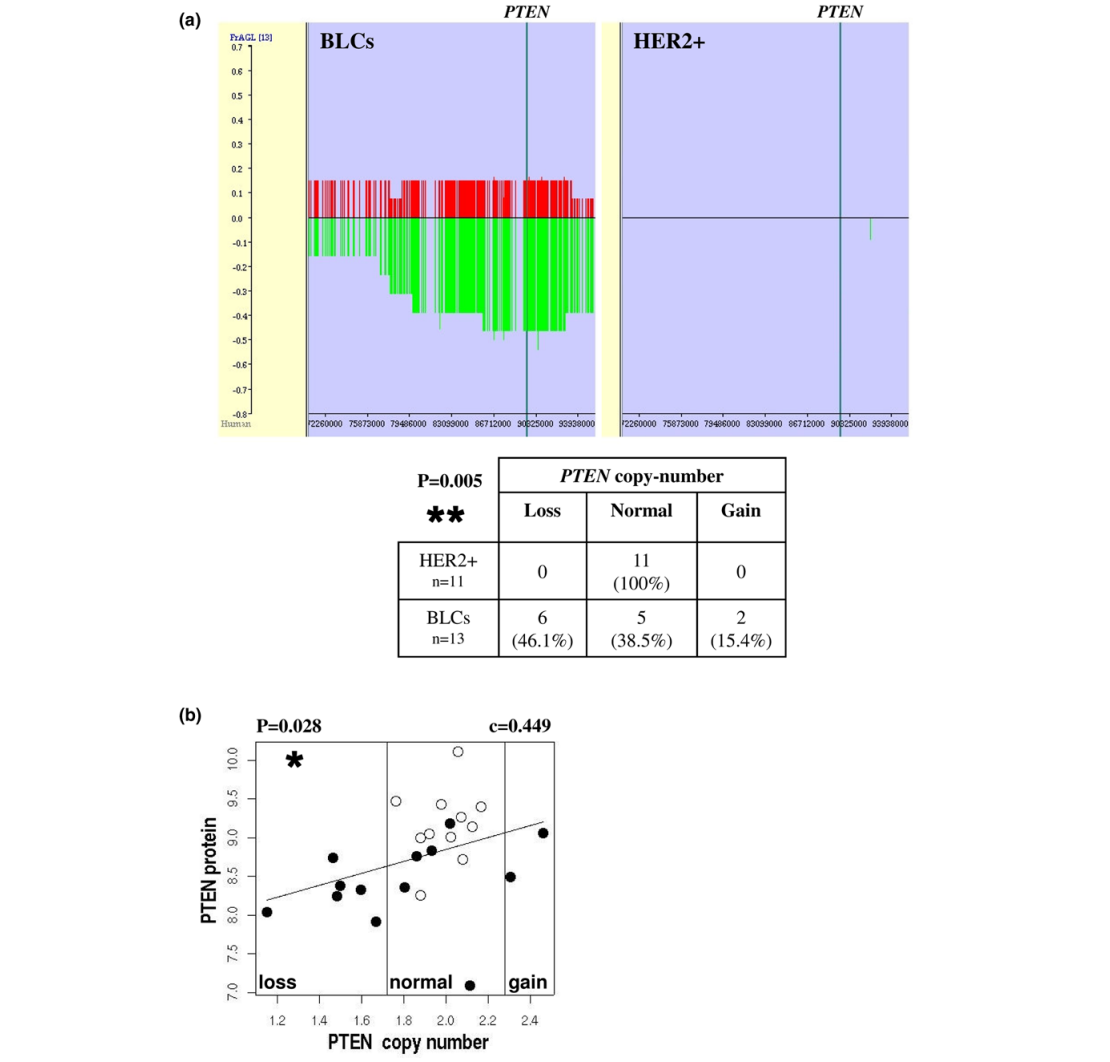
**Loss of *phosphatase and tensin homolog deleted on chromosome 10 (PTEN) *DNA copy-number (CN) in human basal-like breast cancers**. (a) Basal-like carcinomas (BLCs) and human epidermal growth factor receptor overexpressing (HER2+) carcinomas behaved differently for *PTEN *CN in a significant manner. Recurrent DNA CN alterations were observed around the *PTEN *gene (between 72,260,000 and 93,93,000 bp of chromosome 10) in BLCs compared with HER2+ carcinomas. Frequencies of genome CN gain (red) and loss (green) were calculated using the FrAGL (Frequency of Amplicon, Gain and Loss) option of VAMP software (Visualisation and Analysis of array-CGH, transcriptome and other Molecular Profiles) [63]. The vertical blue bar represents *PTEN *position from 89,613,175 to 89,718,511 bp. Percentages of tumours with loss, normal or gain of *PTEN *CN are presented within the two populations in the table. p value is shown (** p < 0.01, fisher exact test). (b) Correlation between PTEN protein level and *PTEN *DNA CN. PTEN protein level was quantified as in Figure 3a. Linear regression, Spearman correlation c and p value (* p < 0.05) are presented. BLCs (solid circles) and HER2+ carcinomas (open circles) are shown. The two vertical black lines (X = 2 ± 0.28) separate loss/normal/gain *PTEN *CN (forceGL parameter: 0.28).

We then examined whether variations in PTEN protein expression could arise from genomic alterations in our BLC population. Genomic DNA isolated from tumours was analysed on SNP arrays. The two populations behaved differently for *PTEN *DNA copy-number (CN) in a significant manner (p = 0.005) (Figure 4a) [see Additional data file 5]. In contrast to the entire HER2+ population exhibiting normal *PTEN *CN, loss of *PTEN *CN was observed in 46.1% (6 of 13) BLCs (Figure 4a) [see Additional data file 5]. Of note is that our BLC population included one *BRCA1 *tumour (c.2501delG *BRCA1 *mutation) which also presented a loss of *PTEN *CN. We noticed that the only double deletion of the *PTEN *gene was observed in a BLC patient with a normal status of *BRCA1 *with the exception of the c.4039A>G polymorphism. We also observed a gain of *PTEN *CN in 2 of 13 BLCs (15.4%) (Figure 4a) [see Additional data file 5] but these two tumours expressed PTEN protein at a level similar to that one in BLCs with normal *PTEN *CN (Figure 4b).

Importantly, *PTEN *CN correlated with PTEN protein level in a significant manner (p = 0.028) in the whole population (Figure 4b). These results suggest that genomic alterations at the *PTEN *locus are directly responsible for low PTEN protein expression in about 50% of BLCs (Figure 4b). Low PTEN protein expression in the other half of BLCs may result from *PTEN *promoter methylation and/or *PTEN *mutation. Although coding mutations of *PTEN *were thought to be rare in breast cancer, *PTEN *nucleotide sequence mutations have recently been detected exclusively in PTEN-null non-hereditary breast cancer [34]. However, we did not detect any *PTEN *mutation in our series of 13 BLCs (data not shown), in agreement with a recent report showing that the rare PTEN mutations observed in breast cancer (2.3%) were restricted to hormone receptor-positive carcinomas [33]. Therefore, low PTEN protein expression in the 50% BLCs with no *PTEN *CN loss may arise from epigenetic modifications.

In addition, by analysing a public data set generated from 42 BLCs and 32 hormone receptors-positive luminal A breast carcinomas [52], we also found a loss of *PTEN *CN, mainly in BLCs, and a correlation between *PTEN *CN and PTEN mRNA in the entire population (p = 3.25 × 10^-7^, c = 0.614, Spearman correlation, data not shown). In conclusion, we demonstrate the presence of genomic alterations at the *PTEN *locus specifically in BLCs. Our findings indicate that alteration of *PTEN *gene is not restricted to BRCA1-associated hereditary tumours (mostly corresponding to a specific basal-like subgroup) as recently suggested [34], but could be extended to the entire BLC population. These genetic modifications may drive to an aberrant PTEN-dependent signalling pathway in the whole BLC population.

### PTEN-dependent activation of Akt in basal-like breast cancer

**Figure 5:**
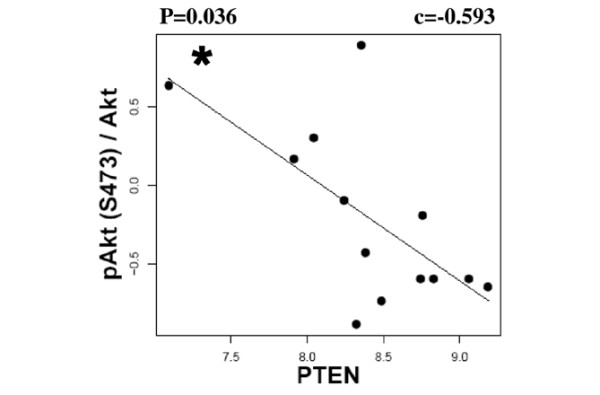
**Phosphatase and tensin homolog deleted on chromosome 10 (PTEN)-dependent activation of Akt in human basal-like breast cancers**. PTEN protein levels are correlated negatively with Akt activity in human basal-like cancer (BLC). Akt activity and PTEN protein levels were measured as in Figures 2c and 3a, respectively. BLCs (solid circles), linear regression, Spearman correlation c and p value (* p < 0.05) are shown.

Low PTEN expression may therefore be responsible for Akt activation in BLCs. Indeed, data obtained by RPPA demonstrated that Akt activity correlated negatively with PTEN expression levels in BLCs (p = 0.036) (Figure 5) but not in HER2+ carcinomas (not shown). Similar conclusions arose from Western blot analysis [see Figure d in Additional data file 4]. Altogether, our data demonstrated a PTEN-dependent activation of Akt in BLCs, consistent with recent work showing higher phospho-Akt levels in PTEN-low compared with PTEN-high breast cancers [33].

We can not rule out the hypothesis that Akt could be activated through multiple mechanisms in BLCs, and not only through low PTEN expression. For example, transcriptomic microarray analysis revealed that the type II inositol polyphosphate-4-phosphatase mRNAs were expressed at significantly lower levels in BLCs compared with HER2+ human tumours (INPP4B reporter 205376_at from Affymetrix; p = 0.0001, data not shown). As INPP4B has been shown to negatively regulate Akt activity [53], its lower expression may represent an alternative pathway for Akt activation in BLCs. However, we could not test this hypothesis at a proteomic level because of the poor quality of the INPP4B antibody available. Mutations of *PIK3CA*, although more frequent in hormone receptor-positive tumours (34.5%) and HER2+ carcinomas (22.7%) occurs in BLCs (8.3%) and could represent another way to activate the PI3K signalling pathway in these tumours [33].

### PI3K but not mTOR inhibition induces apoptosis in basal-like cell lines

**Figure 6:**
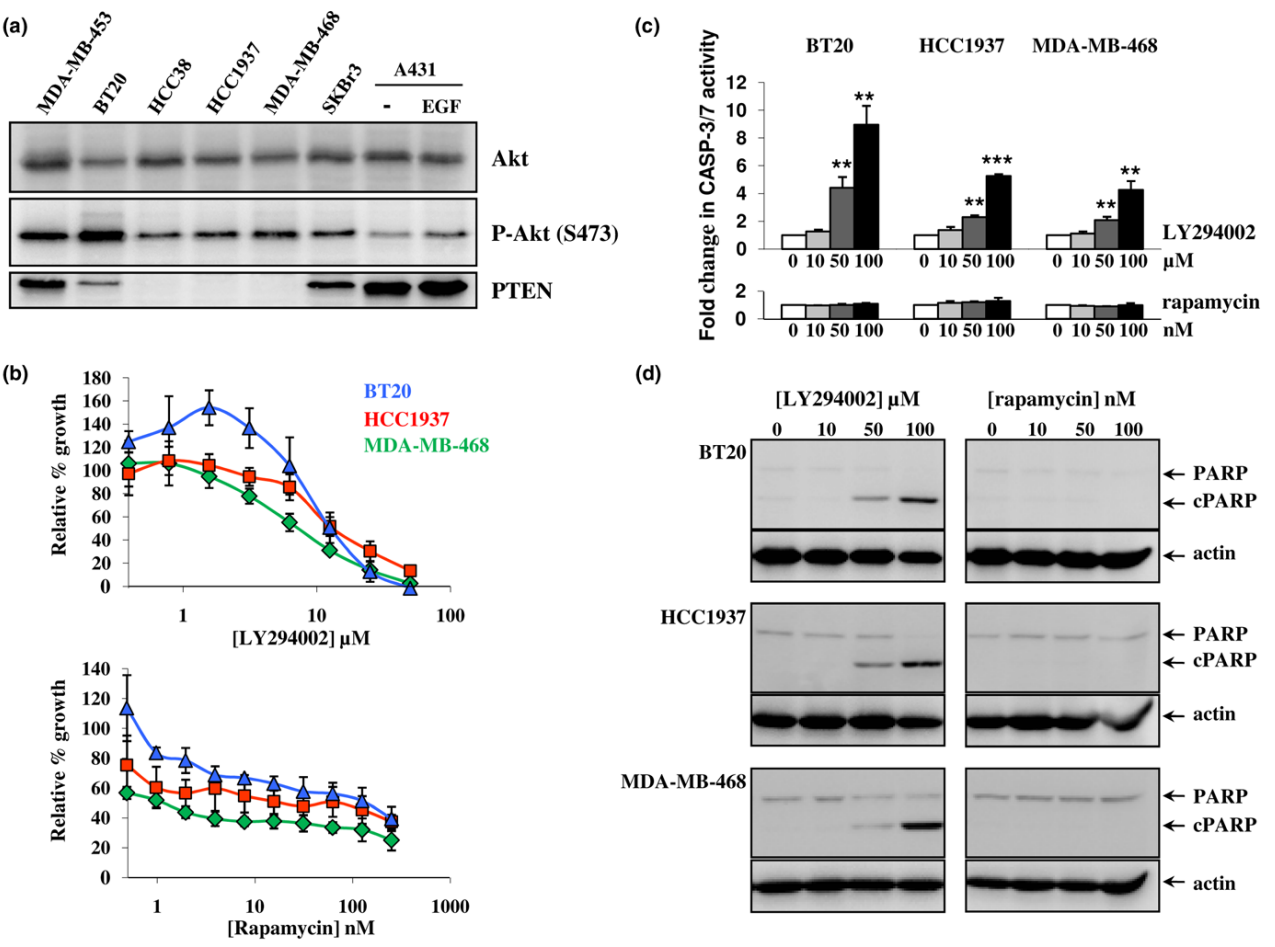
**Phosphatidylinositol 3-kinase (PI3K) and mTOR inhibitors inhibit basal-like cell line proliferation whereas apoptosis is induced only by PI3K inhibition**. (a) Akt activation is associated with low/lack of phosphatase and tensin homolog deleted on chromosome 10 (PTEN) expression in human basal-like cell lines. The expression of Akt, phospho-Akt (S473) and PTEN were analysed by Western blotting in four basal-like (BT20, HCC38, HCC1937 and MDA-MB-468), one human epidermal growth factor receptor overexpressing (HER2+) (SKBr3) and one luminal (MDA-MB-453) human breast cell lines as well as in epidermal growth factor stimulated (EGF) or not (-) A431 cells. (b) PI3K and mTOR inhibition induce cell growth arrest of basal-like cell lines. BT20 (blue triangle), HCC1937 (red square) and MDA-MB-468 (green diamond) cells were exposed continuously for seven days to increasing concentrations of LY294002 (upper panel) or rapamycin (lower panel). Growth was assessed by 3-(4,5-dimethylthiazol-2-yl)-2,5 diphenyltetrazolium bromide (MTT) dye conversion and presented as the percentage of control cell growth inhibition obtained from DMSO-treated cells. The x axes represent logarithmic transformed concentration of drugs. (c,d) The inhibition of PI3K, but not mTOR, induces apoptosis in basal-like cell lines. BT20, HCC1937 and MDA-MB-468 were exposed to varying concentrations of LY294002 (0 to 100 μM) or rapamycin (0 to 100 nM) for 24 hours and apoptosis was detected by measuring (c) caspase3/7 activity and the (d) cleavage of PARP (cPARP). (c) Caspase 3/7 activity was normalised by caspase 3/7 activity from vehicle-treated cells. (d) Actin was used as a loading control. The data represented the (b,c) average of three separate experiments performed in triplicates or are representative of (a,d) three separate experiments. Error bars represent standard deviation and p values (** p < 0.01, *** p < 0.001) were calculated by using Student's *t *test.

Akt activity was examined by Western blotting in four human basal-like cell lines (BT20, HCC38, HCC1937 and MDA-MB-468), one HER2+ (SKBr3) and one luminal (MDA-MB-453) human breast cell lines as well as in an epidermoid carcinoma cell line (A431) for a control (Figure 6a). Akt was phosphorylated indicating that PI3K pathway was activated in all breast cell lines analyzed (Figure 6a). PTEN was weakly expressed (BT20) or not detectable specifically in basal-like cell lines (Figure 6a). We noticed highest levels of Akt phosphorylation in MDA-MB-453 and BT20 (Figure 6a), and this may result from the mutation of the PI3K catalytic subunit (PIK3CA) reported in these two cell lines [24,33]. *PTEN *has been shown to be mutated in MDA-MB-468 [33]. Therefore, similar results were obtained from human biopsies and cell lines revealing an activation of Akt associated with a low/lack expression of PTEN in the basal-like population.

We then investigated whether the inhibition of the PI3K pathway altered proliferation and apoptosis of basal-like cell lines. First, we examined the growth inhibition response of three basal-like cell lines (BT20, HCC1937 and MDA-MB-468) treated with the PI3K inhibitor LY294002 or the mTOR inhibitor rapamycin. Exposure to LY294002 induced an inhibition of the proliferation for all three cell lines with a lower IC_50 _for MDA-MB-468 (IC_50 _= 7.6 ± 1.4 μM) compared with HCC1937 (IC_50 _= 14.5 ± 3.8 μM) and BT20 (IC_50 _= 13.3 ± 2.8 μM) (Figure 6b). The IC_50 _were in the same range than those obtained previously for MDA-MB-468 (IC_50 _= 9.5 μM) [54] and for other breast cell lines (2 to 20 μM LY294002) [55]. MDA-MB-468 cells were the most sensitive cells to LY294002 in agreement with the idea that PTEN mutation render cells more sensitive to growth inhibition by that inhibitor [33]. Exposure to rapamycin led to a growth inhibition that was not complete. The IC_50 _for rapamycin were not reached for HCC1937 and BT20 cell lines. MDA-MB-468 cells were the most sensitive cells to rapamycin with an IC_50 _= 1.2 ± 0.5 nM (Figure 6b). Similar data have been published previously for MDA-MB-468 cells (IC_50 _= 1 nM) [56,57].

We next evaluated whether the growth inhibition resulted from apoptosis. Basal-like cell lines were treated with concentrations of inhibitors used to induce apoptosis, that is 50 to 100 μM LY294002 or 100 nM rapamycin [55-57]. Apoptosis was analysed 24 hours later by measuring casapase 3/7 activity (Figure 6c) and PARP cleavage (Figure 6d). In contrast to rapamycin, LY294002 treatment-induced apoptosis in all basal-like cell lines as judged by a rapamycin dose-dependent increased of caspase 3/7 activity (Figure 6c) and PARP cleavage (Figure 6d). These data are in agreement with a recent paper showing that LY294002 treatment, but not rapamycin, induced apoptosis in other breast cell lines [55]. It is likely that rapamycin inhibited basal-like cell proliferation by arresting the cell cycle in the G1 phase as reported for other breast cell lines [56].

In conclusion, exposure of basal-like cell lines to PI3K or mTOR inhibitors led to cell growth arrest but apoptosis was only observed in cells treated with LY294002. The inhibition of PI3K will directly affect Akt activity, which is involved in cell death and survival through several targets such as Bad, whereas the inhibition of mTOR, which acts downstream of Akt, is expected to inhibit proliferation but not apoptosis [28]. Moreover, the inhibition of mTOR may contribute to an unexpected activation of Akt through a negative feedback loop [58,59]. In order to bypass feedback loops, it may be more efficient to target PI3K or Akt than inhibiting mTOR. In contrast to LY294002, which broadly acts on the majority of PI3Ks and other related kinases [60], inhibitors of specific PI3K isoforms were recently identified [61]. In breast cell lines, PTEN loss was shown to sensitise to p110 beta inhibitors, a ubiquitously expressed class IA PI3K isoform [61]. Moreover, the inhibition of p110 beta was shown to block the tumourigenesis caused by *PTEN *loss in prostate [62]. Although further work is needed, these observations suggest that p110 beta may represent an attractive target for the treatment of patients with low PTEN expressing carcinomas such as BLCs.

## Conclusion

Significant differences of protein expression patterns were observed between BLCs and HER2+ carcinomas, two types of highly proliferative breast cancers. Our data demonstrate that: the PI3K pathway is activated in BLCs and, to a higher extent than in HER2+ carcinomas, is known to have up-regulated Akt and mTOR activities; BLCs express less PTEN compared with HER2+ carcinomas and normal tissues; genomic alterations at the *PTEN *locus are specifically found in BLCs; low PTEN expression in BLCs is associated with lost of *PTEN *DNA CN; Akt activity is dependent of PTEN expression in BLCs; similarly to human biopsies, basal-like breast cell lines exhibit low PTEN expression and activated Akt; PI3K or mTOR inhibition induced growth arrest in basal-like cell lines; PI3K inhibition, but not mTOR inhibition, induced apoptosis of basal-like cell lines; and finally that RPPA is a powerful quantitative tool for proteomic analysis and to examine signalling pathways in human tumours. Our study provides insight into the molecular pathology of BLCs with therapeutic implications and encourages the targeting of key players within the PI3K pathway, such as specific PI3K/Akt isoforms for the management of patients with poor prognosis BLC.

## Abbreviations

AFA: alcohol, formalin and acetic acid; BLC: basal-like breast carcinoma; BSA: bovine serum albumin; CK: cytokeratin; CN: DNA copy number; EGFR: epidermal growth factor receptor; ER: oestrogen receptor; HER2: human epidermal growth factor receptor 2; HER2+ carcinomas: HER2 overexpressing carcinomas; HES: haematoxylin-eosin-safran; IC_50_: inhibition concentration 50%; mTOR: mammalian target of rapamycin; MTT: 3-(4,5-dimethylthiazol-2-yl)-2,5 diphenyltetrazolium bromide; PBS: phosphate buffered saline; PI3K: phosphatidylinositol 3-kinase; PIK3CA: PI3K p110 subunit alpha; PIP3: phosphatidylinositol-3,4,5-trisphosphate; PR: progesterone receptor; PTEN: phosphatase and tensin homolog deleted on chromosome 10; RPPA: reverse phase protein array; SNP: single nucleotide polymorphism; TBS: tris buffer saline; TBST: TBS + 0.1% tween-20; TBST-BSA: TBST + 5% BSA; TMA: tissue microarray.

## Competing interests

The authors declare that they have no competing interests.

## Authors' contributions

BM developed and performed the RPPA experiments and analyses. VM made the *in vitro *functional analyses and PTEN sequencing. EG, GR, PH, PG and EB contributed to statistical and bioinformatics analyses and development of informatics tools. AVS and IL collected, selected and provided human samples including DNA, RNA and biopsies. AVS and MK were involved in immunohistochemistry and TMA studies. VM, FD and AT performed the experiments in human breast cell lines. TD conceived the study design and performed the Western blot experiments from human samples. AVS, BM, FC, GCT, JPT and JAH participated in the design of the study. BM, VM, AVS, MK, FC, GCT, MHS, JAH and TD contributed to the biological interpretations of data. AVS, MHS and JPT provided expertise in clinical breast oncology. MHS and GR made the analysis of PTEN copy number from Affymetrix SNP data. BM and TD were responsible of manuscript editing. VM, EG, GR, AVS, MK, IL, MHS and JAH contributed to the preparation and corrections of the manuscript. All authors read and approved the final manuscript.

## Supplementary Material

Additional file 1 A PDF containing figures showing the expression of HER2 measured by Western blotting and its correlation with RPPA data. Figure a illustrates the expression of total HER2 protein expression measured by Western blotting in human BLCs and HER2+ carcinomas. P value (*** p < 0.001) is represented (Mann-Whitney test). Figure b illustrates the correlation between RPPA and Western-blotting analysis for HER2 protein expression. Protein expressions are logarithmic transformed and illustrated by box plots with p values (Mann-Whitney test). Outliers are shown within the BLCs (solid circles) and HER2+ carcinomas (open circles) populations. The correlations are estimated using the Spearman correlation test (c) from logarithmic transformed values. Linear regression and p values are indicated. BLCs (solid circles) and HER2+ carcinomas (open circles) are shown. The significant p values are represented by stars (* p < 0.05, ** p < 0.01, *** p < 0.001). Click here for file

Additional file 2 A PDF containing figures showing Akt activation in basal-like breast cancer measured by Western blotting and its correlation with RPPA data. Figures a, b and c illustrate the expression of total Akt, the phosphorylated/active form of Akt (phospho-Akt (S473)), and the activity of Akt determined as the 'phospho/total' ratio, respectively, in human BLCs and HER2+ carcinomas. Figures d and e show the correlation between RPPA and Western blotting data for Akt and phospho-Akt protein expressions, respectively. Protein expressions are logarithmic transformed and illustrated by box plots with p values (Mann-Whitney test). Outliers are shown within the BLCs (solid circles) and HER2+ carcinomas (open circles) populations. The correlations are estimated using the Spearman correlation test (c) from logarithmic transformed values. Linear regression and p values are indicated. BLCs (solid circles) and HER2+ carcinomas (open circles) are shown. The significant p values are represented by stars (* p < 0.05, ** p < 0.01, *** p < 0.001). Click here for file

Additional file 3 A PDF containing a figure showing that the active form of Akt is detected in tumour cells within the biopsies by immunohistochemistry. Expression and localisation of phospho-Akt (S473), and hence activated Akt, was analysed on TMA in BLCs and HER2+ carcinomas. Phospho-Akt showed low, medium and high expression depending on the tumour samples. Phospho-Akt is preferentially expressed in tumour cells. It is located in the cytoplasm and at the plasma membrane particularly in tumour cells with strong staining. All photomicrographs are of the same 40× magnification (scale bar, 20 μm). Click here for file

Additional file 4 A PDF containing a figure that validates the PTEN-dependent activation of Akt observed by RPPA in basal-like cancer with Western blot technology. Figure a shows PTEN protein level. Data are representative of two separate experiments. Figure b indicates the correlation between RPPA and Western blotting analysis for PTEN protein expression. Figure c exhibits the correlation between PTEN protein measured by Western blotting and PTEN messenger (probeset 225363_at). Figure d shows that PTEN protein levels correlates negatively with Akt activity within the BLC population (solid circles). Akt activity and PTEN protein levels were measured by Western blotting as in Figure a to c in Additional file 2 and as in Figure a in Additional file 4, respectively. Protein expressions are logarithmic transformed and illustrated by box plots with p values (Mann-Whitney test). Outliers are shown within the BLCs (solid circles) and HER2+ carcinomas (open circles) populations. The correlations are estimated using the Spearman correlation test (c) from logarithmic transformed values. Linear regression and p values are indicated. BLCs (solid circles) and HER2+ carcinomas (open circles) are shown. The significant p values are represented by stars (* p < 0.05, ** p < 0.01, *** p < 0.001). Click here for file

Additional file 5 A PDF containing a figure that illustrates recurrent genomic alterations around PTEN locus in human basal-like breast cancers. Genomic analysis using the VAMP software (Visualization and Analysis of array-CGH, transcriptome and other Molecular Profiles) [60] around the PTEN gene (between 80,676,000 and 98,604,000 bp of chromosome 10) are shown for all the 13 BLCs (upper panel) and the 11 HER2+ tumours (lower panel). Each row represents one tumour profile. Gains are in red, losses in green and absence of alterations in yellow. The vertical blue bars represent the position of PTEN from 89,613,175 to 89,718,511 bp. Click here for file
