# Supplementary figures and images for: Frequent PTEN genomic alterations and activated phosphatidylinositol 3-kinase pathway in basal-like breast cancer cells
Source: Breast Cancer Res. 2008 Dec 3;10(6):R101. doi: 10.1186/bcr2204 (PMC2656897; doi:10.1186/bcr2204)

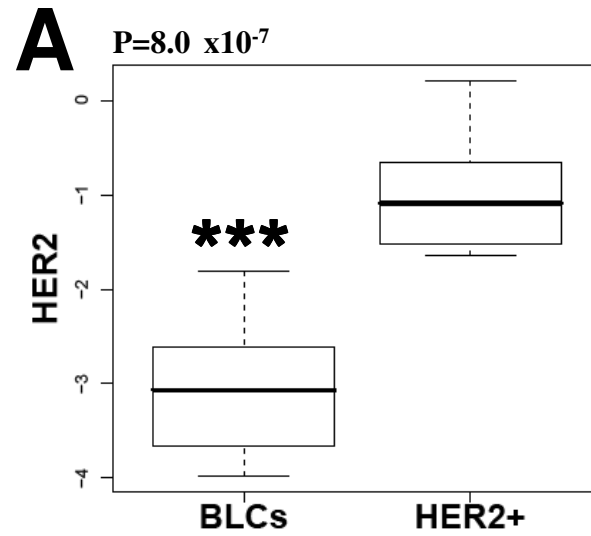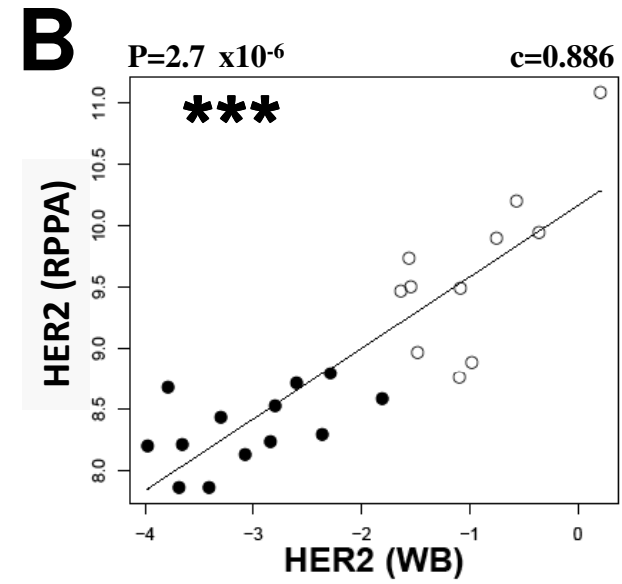

Supplement: Additional file 1 — A PDF containing figures showing the expression of HER2 measured by Western blotting and its correlation with RPPA data. Figure a illustrates the expression of total HER2 protein expression measured by Western blotting in human BLCs and HER2+ carcinomas. P value (*** p < 0.001) is represented (Mann-Whitney test). Figure b illustrates the correlation between RPPA and Western-blotting analysis for HER2 protein expression. Protein expressions are logarithmic transformed and illustrated by box plots with p values (Mann-Whitney test). Outliers are shown within the BLCs (solid circles) and HER2+ carcinomas (open circles) populations. The correlations are estimated using the Spearman correlation test (c) from logarithmic transformed values. Linear regression and p values are indicated. BLCs (solid circles) and HER2+ carcinomas (open circles) are shown. The significant p values are represented by stars (* p < 0.05, ** p < 0.01, *** p < 0.001). [file bcr2204-S1.pdf]

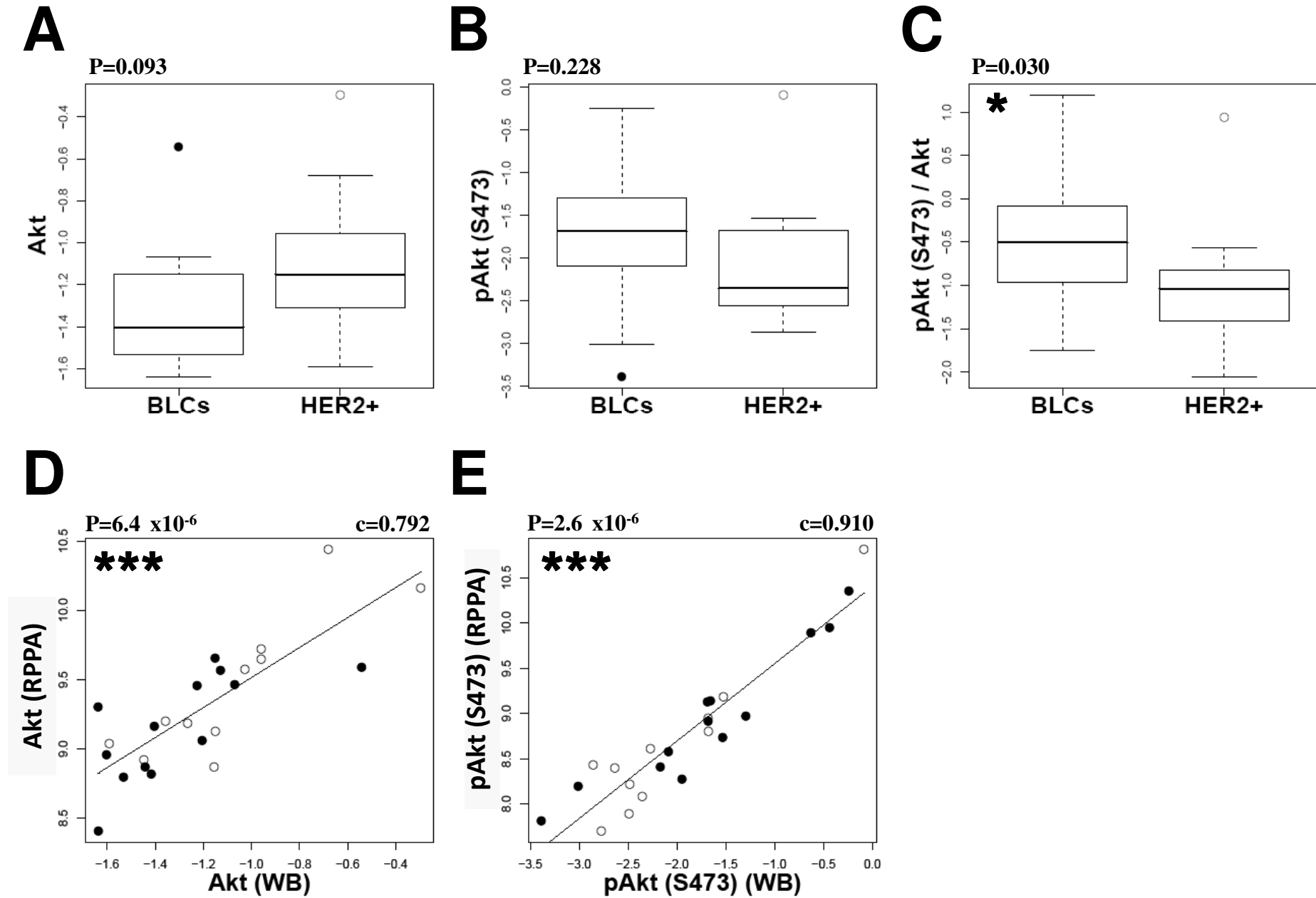

Supplement: Additional file 2 — A PDF containing figures showing Akt activation in basal-like breast cancer measured by Western blotting and its correlation with RPPA data. Figures a, b and c illustrate the expression of total Akt, the phosphorylated/active form of Akt (phospho-Akt (S473)), and the activity of Akt determined as the 'phospho/total' ratio, respectively, in human BLCs and HER2+ carcinomas. Figures d and e show the correlation between RPPA and Western blotting data for Akt and phospho-Akt protein expressions, respectively. Protein expressions are logarithmic transformed and illustrated by box plots with p values (Mann-Whitney test). Outliers are shown within the BLCs (solid circles) and HER2+ carcinomas (open circles) populations. The correlations are estimated using the Spearman correlation test (c) from logarithmic transformed values. Linear regression and p values are indicated. BLCs (solid circles) and HER2+ carcinomas (open circles) are shown. The significant p values are represented by stars (* p < 0.05, ** p < 0.01, *** p < 0.001). [file bcr2204-S2.pdf]

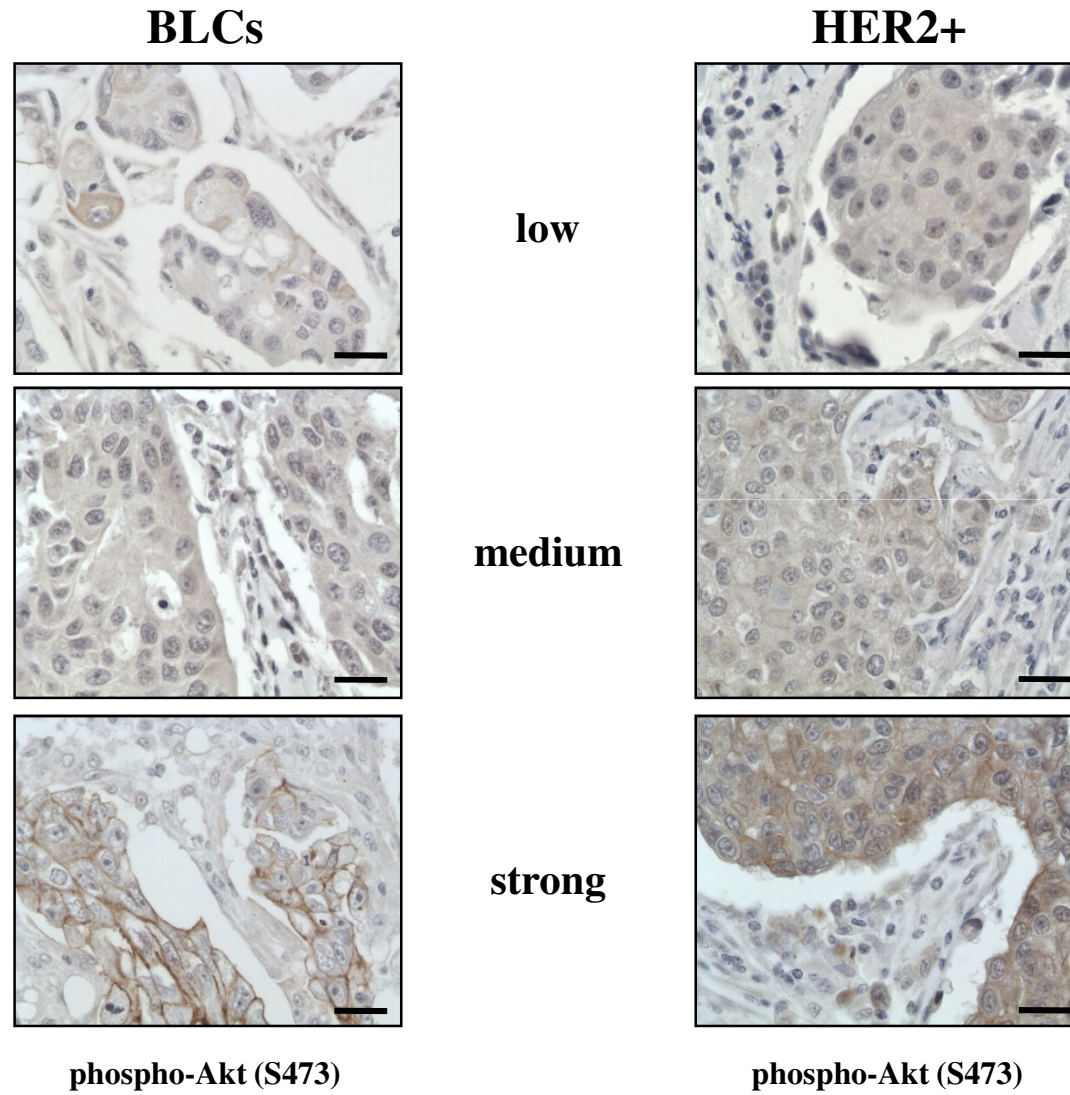

Supplement: Additional file 3 — A PDF containing a figure showing that the active form of Akt is detected in tumour cells within the biopsies by immunohistochemistry. Expression and localisation of phospho-Akt (S473), and hence activated Akt, was analysed on TMA in BLCs and HER2+ carcinomas. Phospho-Akt showed low, medium and high expression depending on the tumour samples. Phospho-Akt is preferentially expressed in tumour cells. It is located in the cytoplasm and at the plasma membrane particularly in tumour cells with strong staining. All photomicrographs are of the same 40× magnification (scale bar, 20 μm). [file bcr2204-S3.pdf]

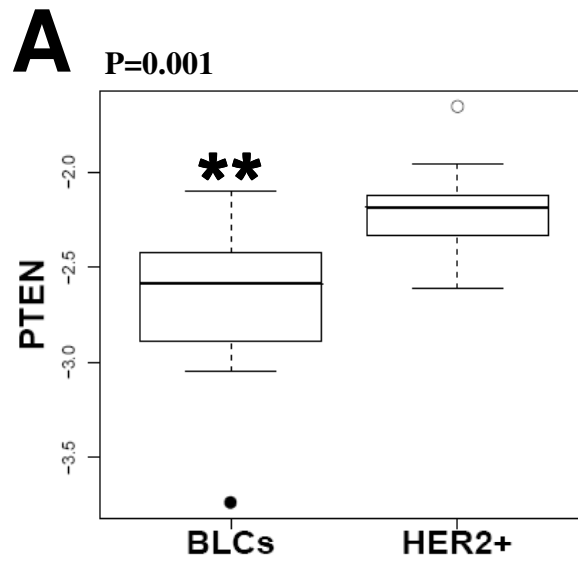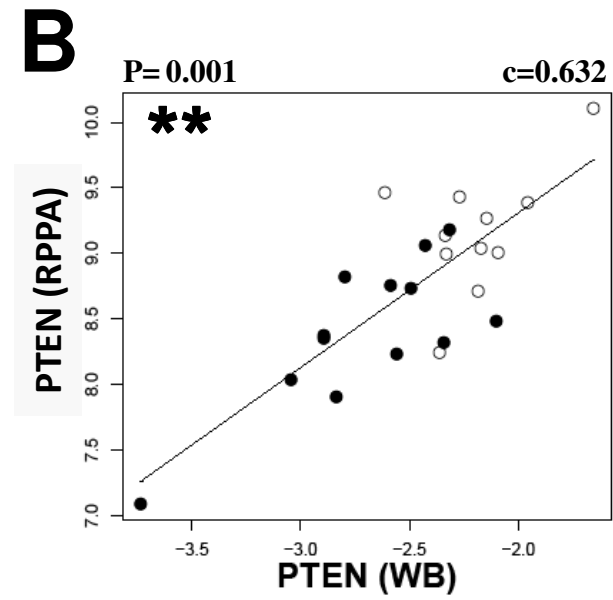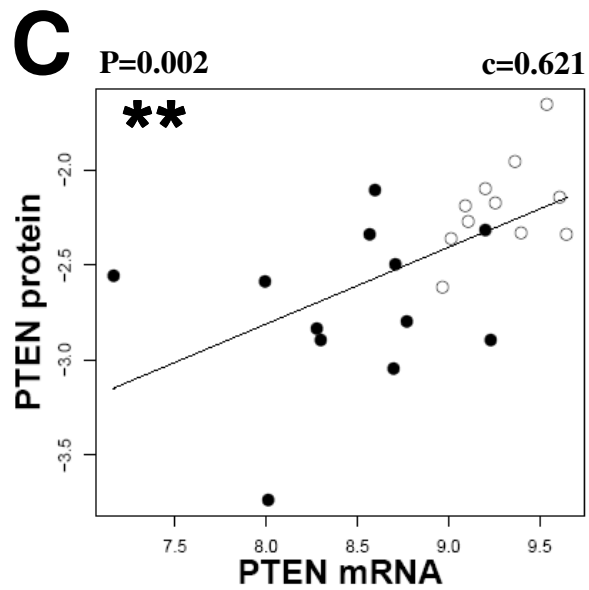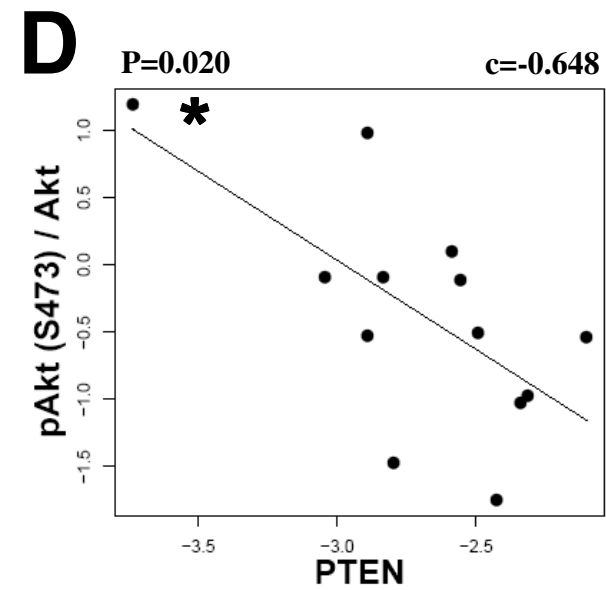

Supplement: Additional file 4 — A PDF containing a figure that validates the PTEN-dependent activation of Akt observed by RPPA in basal-like cancer with Western blot technology. Figure a shows PTEN protein level. Data are representative of two separate experiments. Figure b indicates the correlation between RPPA and Western blotting analysis for PTEN protein expression. Figure c exhibits the correlation between PTEN protein measured by Western blotting and PTEN messenger (probeset 225363_at). Figure d shows that PTEN protein levels correlates negatively with Akt activity within the BLC population (solid circles). Akt activity and PTEN protein levels were measured by Western blotting as in Figure a to c in Additional file 2 and as in Figure a in Additional file 4, respectively. Protein expressions are logarithmic transformed and illustrated by box plots with p values (Mann-Whitney test). Outliers are shown within the BLCs (solid circles) and HER2+ carcinomas (open circles) populations. The correlations are estimated using the Spearman correlation test (c) from logarithmic transformed values. Linear regression and p values are indicated. BLCs (solid circles) and HER2+ carcinomas (open circles) are shown. The significant p values are represented by stars (* p < 0.05, ** p < 0.01, *** p < 0.001). [file bcr2204-S4.pdf]

BLCs

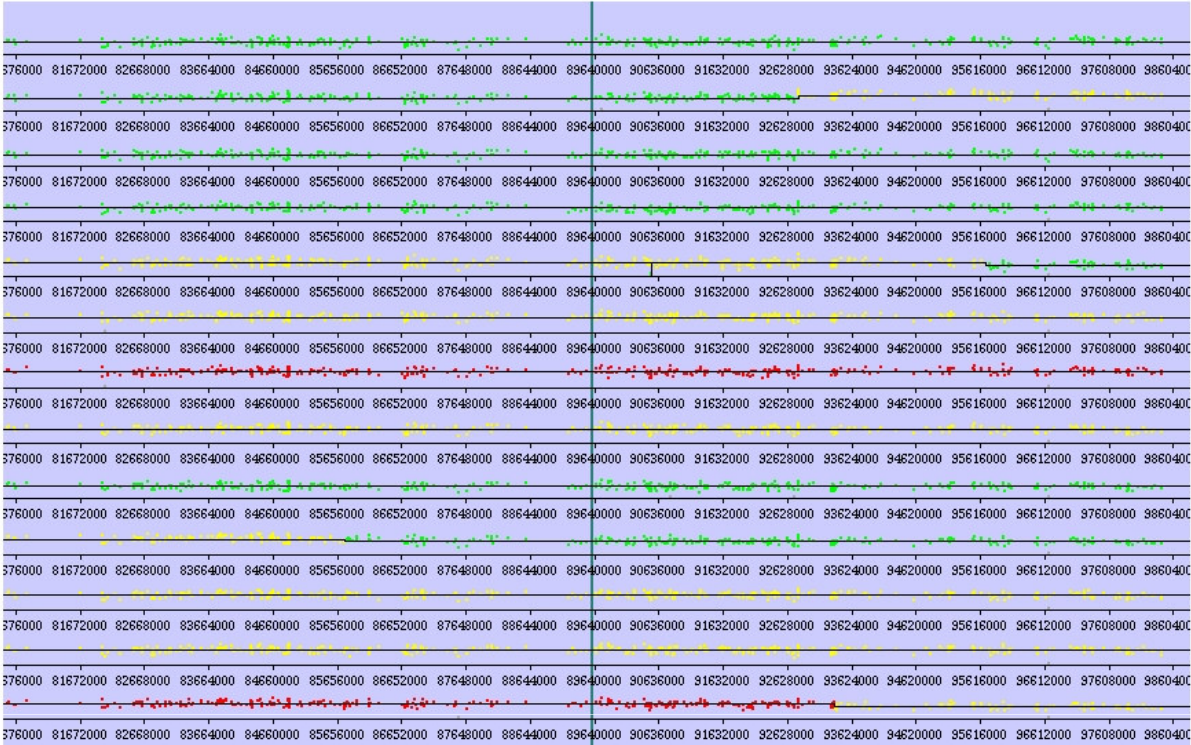

*PTEN*

HER2+

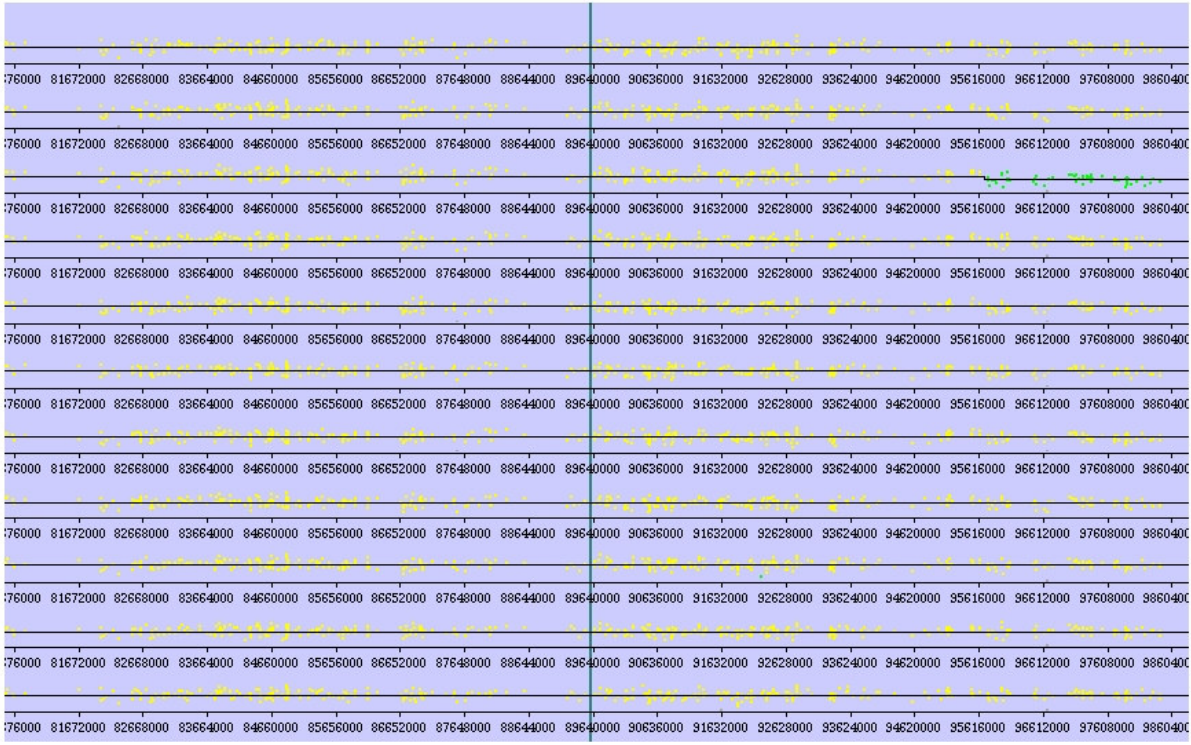

*PTEN*

Supplement: Additional file 5 — A PDF containing a figure that illustrates recurrent genomic alterations around PTEN locus in human basal-like breast cancers. Genomic analysis using the VAMP software (Visualization and Analysis of array-CGH, transcriptome and other Molecular Profiles) [60] around the PTEN gene (between 80,676,000 and 98,604,000 bp of chromosome 10) are shown for all the 13 BLCs (upper panel) and the 11 HER2+ tumours (lower panel). Each row represents one tumour profile. Gains are in red, losses in green and absence of alterations in yellow. The vertical blue bars represent the position of PTEN from 89,613,175 to 89,718,511 bp. [file bcr2204-S5.pdf]
